# Modeling vegetation greenness and its climate sensitivity with deep‐learning technology

**DOI:** 10.1002/ece3.7564

**Published:** 2021-05-02

**Authors:** Zhiting Chen, Hongyan Liu, Chongyang Xu, Xiuchen Wu, Boyi Liang, Jing Cao, Deliang Chen

**Affiliations:** ^1^ College of Urban and Environmental Sciences and MOE Laboratory for Earth Surface Processes Peking University Beijing China; ^2^ Faculty of Geographical Sciences Beijing Normal University Beijing China; ^3^ August Röhss Chair Department of Earth Sciences University of Gothenburg Gothenburg Sweden

**Keywords:** climate change, climate sensitivity, deep learning, long short‐term memory network, vegetation greenness, vegetation–climate relationship

## Abstract

Climate sensitivity of vegetation has long been explored using statistical or process‐based models. However, great uncertainties still remain due to the methodologies’ deficiency in capturing the complex interactions between climate and vegetation. Here, we developed global gridded climate–vegetation models based on long short‐term memory (LSTM) network, which is a powerful deep‐learning algorithm for long‐time series modeling, to achieve accurate vegetation monitoring and investigate the complex relationship between climate and vegetation. We selected the normalized difference vegetation index (NDVI) that represents vegetation greenness as model outputs. The climate data (monthly temperature and precipitation) were used as inputs. We trained the networks with data from 1982 to 2003, and the data from 2004 to 2015 were used to validate the models. Error analysis and sensitivity analysis were performed to assess the model errors and investigate the sensitivity of global vegetation to climate change. Results show that models based on deep learning are very effective in simulating and predicting the vegetation greenness dynamics. For models training, the root mean square error (RMSE) is <0.01. Model validation also assure the accuracy of our models. Furthermore, sensitivity analysis of models revealed a spatial pattern of global vegetation to climate, which provides us a new way to investigate the climate sensitivity of vegetation. Our study suggests that it is a good way to integrate deep‐learning method to monitor the vegetation change under global change. In the future, we can explore more complex climatic and ecological systems with deep learning and coupling with certain physical process to better understand the nature.

## Graphical abstract

Our study is first of its kind aimed to predict ecosystem dynamics using deep learning technology. We developed a method to quantify sensitivity of vegetation greenness to climate change in the context of deep learning, which contribute to the application of deep learning in global change ecology. Our biome‐specific sensitivity of vegetation greenness to climate change can be well explained

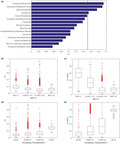

## 1 INTRODUCTION

In the context of global change, terrestrial ecosystems are facing severe challenges. More and more studies have shown that climate change has affected the vegetation greenness and distribution pattern (Connor et al., 2018; Forzieri et al., 2017; Gottfried et al., 2012; Keenan & Riley, 2018; Pearson et al., 2013), and in return, changes in vegetation greenness and distribution pattern provide feedbacks to climate systems through energy fluxes process (Forzieri et al., 2017; Pearson et al., 2013; Xu et al., 2013). It is critical to reveal the complicated relationship between climate and vegetation to better understand the climate feedbacks, ecosystem health, and sustainable development. Accurately predicting the effect of future climate change on vegetation is also one of the major challenges in global change ecology (Allen et al., 2010). However, the ability to reveal the climate–vegetation relationship and project the future is limited by model performances and uncertainties in complex social–ecological systems (Bonan & Doney, 2018; Friedlingstein et al., 2014; Mahowald et al., 2016; McDowell et al., 2016; Rineau et al., 2019). It is necessary to develop new generation models to achieve better vegetation monitoring and ecosystem management.

Scientists have established many vegetation–climate‐related models in recent years. There are two main types: statistical models and ecological process‐based models. The typical statistical relation‐based models, such as Miami model (Lieth, 1975) and Thornthwaite memorial model, can predict the impact of climate on vegetation by establishing the correlation between climate factors and vegetation production. This type of model is simple in form and easy to perform, thus has been widely used in different regions (Stephan et al., 2008). However, models’ errors are relatively large and predictive ability is limited. Another type of models is based on the ecological process, such as the Century (Parton et al., 1993), TEM (McGuire et al., 1997), BIOME‐BGC (Running & Hunt, 1993), and some dynamic vegetation models such as LPJ‐DVGM, LPJ‐GUESS, and IBIS. These models comprehensively consider the process of material and energy exchange between vegetation and environment, promote the mechanism research of the interaction between ecological process and climate change, and have been widely used in modern vegetation dynamic models. The model processes are very complex with large number of variables, which makes it somewhat difficult to be generalized on a global and long‐time scale.

Unprecedented development of big data and information technology provides us with exciting opportunities to explore complex ecosystem issues (Reichman et al., 2011; Reichstein et al., 2019). Many tools in artificial intelligence (AI), particularly machine learning, have been applied to the analysis of earth sciences, especially for making accurate predictions from data (Bergen et al., 2019; Gómez‐Chova et al., 2015; Pearson et al., 2013; Reichstein et al., 2019; Zhu et al., 2017). However, traditional machine learning methods have inherent limitations, that is, the ability to analyze system behaviors with the coupling of time and space is still insufficient (Reichstein et al., 2019). In recent years, the development of deep‐learning technology has solved this problem to a large extent. Deep learning is a multi‐layer representation learning method that allows computers to learn from experiences (LeCun et al., 2015); this technology is at the core of big data analysis and has achieved remarkable success in computer vision, speech recognition (Zhu et al., 2017).

Deep‐learning technology is a branch of machine learning and refers to an algorithm that uses artificial neural networks (ANNs) as the framework for representation learning (LeCun et al., 2015). Through the calculation of the depth of the hidden layers, simple features are mapped to the output through additional layers of more abstract features. And the ability to learn from data further makes deep‐learning algorithm different and powerful. As a result, the complex relationship between the dependent and independent variables can be better mined to improve the accuracy of the model simulation and projection and help us better understand behavior of complex systems (Goodfellow et al., 2016).

Deep learning has been developed rapidly in recent years due to its high flexibility and performance. However, its application in ecology is still in the infancy (Christin et al., 2019; Reddy & Prasad, 2018). Most ecological researches relevant to deep‐learning method are for species identification and classification (Ferreira et al., 2020; Kiskin et al., 2020; Tabak et al., 2019; Wäldchen & Mäder, 2018). Other applications of deep learning in ecology include behavior studies (Browning et al., 2017), statistical downscaling and blending of remote sensing images (Reichstein et al., 2019; Vandal et al., 2018), and ecosystem modeling (Chen et al., 2016; Reddy & Prasad, 2018). Due to the development of big data and automatic monitoring, it is easy and capable to accumulate a large amount of data nowadays, and deep learning proves to be efficient in dealing with huge data and accurate classification and prediction, and with great potential due to its high accuracy and flexibility, especially for dynamic time series modeling as well as for complex relations among variables coupling both time and space scales. However, no previous studies have been carried out to model the vegetation–climate relationship with deep‐learning methods and our study is first of its kind aimed to model and predict ecosystem dynamics with deep learning.

Here, we modeled vegetation dynamics driven by climate factors with deep‐learning technology to achieve accurate vegetation monitoring and investigate the complex relationship between climate and vegetation based on long short‐term memory (LSTM) network. We selected the normalized difference vegetation index (NDVI), derived from the third generation of the Global Inventory Modeling and Mapping System (i.e., GIMMS NDVI 3g), to represent vegetation greenness. A total of 34 years (1982–2015) of monthly NDVI data are taken as dependent variable. The corresponding monthly temperature and precipitation from the Climatic Research Unit product (CRU TS 4.01) are used as independent variables to establish the global gridded climate–vegetation models. Then, we performed sensitivity analysis to investigate the impact of temperature and precipitation on global vegetation. The aim of this paper is to introduce the deep‐learning method to vegetation dynamics modeling and investigate the sensitivity of vegetation to climate change, to better monitor the future ecosystem change.

## 2 MATERIALS AND METHODS

### 2.1 Datasets collection and annotation

We used the NDVI to represent vegetation greenness level. NDVI is widely used in dynamic vegetation monitoring (Eastman et al., 2013; Guo et al., 2017; Kariyeva & Van, 2011; Walker et al., 2012; Xu & Guo, 2014), and it can represent the physiological functions of vegetation and the greenness level in an area better than other indicators (Pinzon & Tucker, 2014a; Tucker et al., 2005). In this paper, we adopted the latest version of the Global Inventory Modeling and Mapping System (GIMMS), the third generation of NDVI data from 1982 to 2015 (https://climatedataguide.ucar.edu/climate‐data/ndvi‐normalized‐difference‐vegetation‐index‐3rd‐generation‐nasagfsc‐gimms), with a temporal resolution of 15 days and a spatial resolution of 0.083° (~8 km). The influences of cloud cover, solar altitude angle, orbital drift and other factors from the data were removed from this dataset (Pinzon & Tucker, 2014b). We processed the data using maximum value compositing (MVC) method (Holben, 1986) to obtain the NDVI. Combined NDVI data with monthly resolution were used to minimize the impact of the atmospheric conditions and clouds on NDVI. The vegetation data were unified to a spatial resolution of 0.5° to match the observational gridded climate data.

Monthly precipitation and temperature data were derived from the Climatic Research Unit (CRU) global climate dataset (http://badc.nerc.ac.uk/browse/badc/cru) based on interpolation of global site observation data. We chose the latest version of the temperature and precipitation data, CRU TS 4.01, with a temporal resolution of a month and a spatial resolution of 0.5°. Data from 1982 to 2015 are selected, and pixel data with invalid values are deleted.

### 2.2 Deep‐learning framework

Deep‐learning frameworks mainly include deep neural networks (DNNs), convolutional neural networks (CNNs), and recurrent neural networks (RNNs) (Goodfellow et al., 2016). We used the long short‐term memory (LSTM) network in this research, which is a variant of recurrent neural network (RNN). RNN is a type of neural network dedicated to processing time series data samples (Graves, 2013). Each layer of the information in a neural network not only outputs to the next layer but also outputs a hidden state, which is used when processing the information to the next sample. The recurrent structure allows the previous information to be continuously saved with a memory effect; thus, it is widely applied in speech recognition and time series modeling.

Long short‐term memory is an improved algorithm based on RNNs that can describe the long‐term dependence of long‐distance time series (Hochreiter & Schmidhuber, 1997). LSTM works similarly to RNNs, with one more cell state parameterized structure added and internal memory to store the previous information, which makes it more powerful in modeling and predicting time series due to its long‐term memory (Gamboa, 2017). Thus, it is a better choice to select LSTM as our model framework since LSTM can better solve long‐term problems and have a faster training effect than other algorithms.

### 2.3 Model training and validation

The basic process of deep‐learning technology is to train a model with a large amount of historical data and input new data to make projections when the model accuracy reaches a certain level. In this study, we modeled long‐term serial vegetation data and climate data for each pixel globally. To determine the optimal structure and parameters of the models, we conducted a preliminary vegetation–climate modeling experiment with 1,000 randomly selected pixels worldwide. By adjusting the parameters of the models and making an experience‐based judgment, we ultimately built models including three layers: two cascading LSTM layers and a fully connected layer. The dimension of each LSTM layer is 75, and the input and output sizes of the models are 2 (temperature and precipitation) and 1 (NDVI), respectively. The model inputs include temperature and precipitation time series data for each grid point. The time step of the models is 6, which means that the network generates the predicted NDVI values for the last 6 months with the six successive months of precipitation and temperature data. We used the error function (MSE) and Adam method to optimize the models (Kingma & Adam, 2014). When the model projection was close to the actual NDVI value within a predefined difference, there was no decrease in the loss curve, the modeling process was completed, and the NDVI value predicted by the model was the output.

The model training effect is dependent on the model training time (*m*). To explore the most suitable training time (*m*) and validation time (*n*) for the models, we randomly selected 1,000 points and analyzed the effects of training years on model performance. Training times ranging from 2, 4, 6 … to 32 years were used to train the models. To ensure sufficient model verification, we ultimately selected 22 years (264 months, 1982–2003) of data to train the model, followed by 12 years (144 months, 2004–2015) for model verification.

For model training, we used the root mean square error (RMSE) to represent the model fitting accuracy. Then, for model validation, we calculated the coefficient of variation (CV) to evaluate the performance of the model (Abdi, 2010). When CV is <15%, the model has better performance and the prediction accuracy is acceptable. When CV is >15%, the model is less effective.

Coefficient of variation is used to measure the deviation of measured data from predicted data, calculated as follows:$\text{CV}=\frac{\sqrt{\frac{1}{n}\sum_{i=1}^{n}{\left(y_{i}‐{\hat{y}}_{i}\right)}^{2}}}{\left|\bar{y}\right|}$where *n* is number of samples, $y_{i}$ is measured data, ${\hat{y}}_{i}$ is predicted data, and $\bar{y}$ is the mean value of measured data.

After removing the missing and invalid values, we finally obtained a total of 53,432 valid pixels globally, and we established 53,432 vegetation–climate models.

### 2.4 Sensitivity analysis

The models established by the deep‐learning method are based on the datasets. We can explore the importance of different independent variables on the dependent variable through sensitivity analysis. In this paper, permutation importance (PI) (Altmann et al., 2010; Strobl et al., 2008) was used to indicate the magnitude of the influence of features on the target variable. The larger the PI value is, the greater the influence of the variable on the target variable. Permutation importance was implemented in Python through the eli5 package (URL: https://pypi.org/project/eli5/). The principle is to keep the other features unchanged in the validation dataset, randomly add noise data to feature X, and obtain a new “mutation” validation set (Strobl et al., 2008). Then, we predicted and scored this new validation dataset and compared the model's performance based on the new validation dataset with that based on the original validation dataset. The larger the difference is, the greater the impact of feature *X* on *Y*.

Since there are only two independent variables (temperature and precipitation) in our study, we calculated the PI of temperature (PIT) and the PI of precipitation (PIP) separately and obtained the temperature and precipitation permutation importance difference (PID) by PIT minus PIP to represent the difference in the sensitivity of vegetation to temperature and precipitation. When PID  ≥ 0.01, vegetation is more sensitive to temperature than it is to precipitation. When PID ≤ −0.01, vegetation is more sensitive to rainfall. When −0.01 < PID < 0.01, vegetation is sensitive to both temperature and rainfall.

### 2.5 Model error analysis

To further explore the sources of model errors, we conducted an error analysis on all the models worldwide. We mainly assessed climate and vegetation factors related to our study, including the following six factors: mean annual temperature (MAT), mean annual precipitation (MAP), temperature change (ΔTMP), interannual variability of temperature (IAT), interannual variability of precipitation (IAP), and interannual variability of vegetation (IAV). The errors of the models are expressed by the CV. Among these factors, the MAT and MAP of each grid point are calculated from CRU climate data averaged over 1982–2015, the ΔTMP is calculated as the average temperature of 2011–2015 minus the average temperature of 1982–1986, the IAT and IAP are calculated from the CV for CRU climate data from 1982 to 2015, and the IAV is calculated from the CV in the NDVI data from 1982 to 2015.

We adopted linear regression method and calculate correlations between the model error CV with the related variables to analyze the relationships between different variables.

In addition, the vegetation variability is related to the land cover type, and we also analyzed the influence of different land cover types on the model performance. The global vegetation classification data are derived from the Moderate Resolution Imaging Spectroradiometer (MODIS) land cover product MOD12C1 (http://glcf.umd.edu/data/lc/). The MOD12C1 data product has an annual resolution and a spatial resolution of 0.05°. We used land cover data from 2012 as the base map with which to divide the global vegetation cover areas. The MOD12C1 data product adopts the International Geosphere‐Biosphere Program scheme, which divides the globe into 17 land use types. In this paper, after removing areas of water and snow, 15 land cover types are used for the analysis. The land cover data were interpolated to a 0.5° resolution to match the NDVI and climate data. The dominant land cover type (the land cover type with the highest proportion within the 0.5° pixel) was used as the land cover type of a given grid point.

## 3 RESULTS

### 3.1 Model performance

**FIGURE 1:**
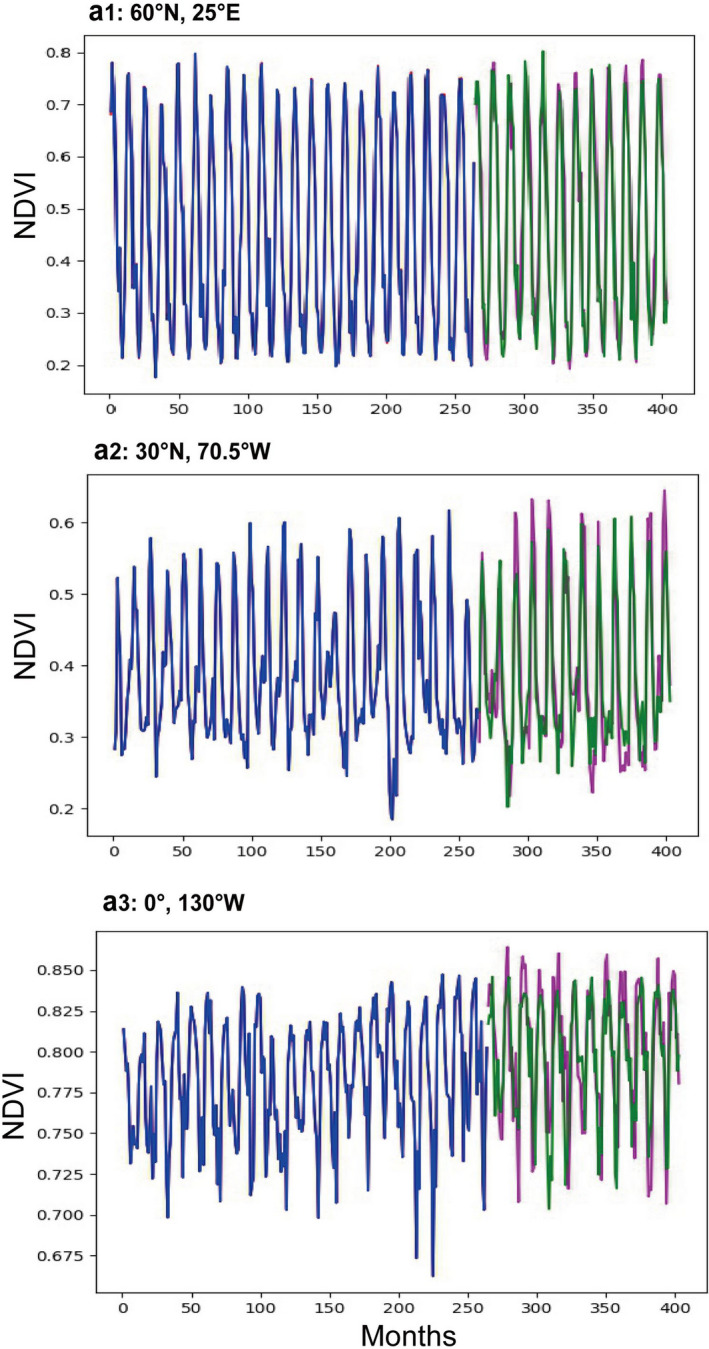
Model simulation and validation based on deep learning. (a1–a3) Three grid models randomly selected across a latitudinal gradient. The first 22 years (264 months) are used to train the models, and the next 12 years (144 months) are used to validate the model accuracy. The red line and purple line represent the original NDVI values, and the blue line and green line represent the model simulations

**FIGURE 2:**
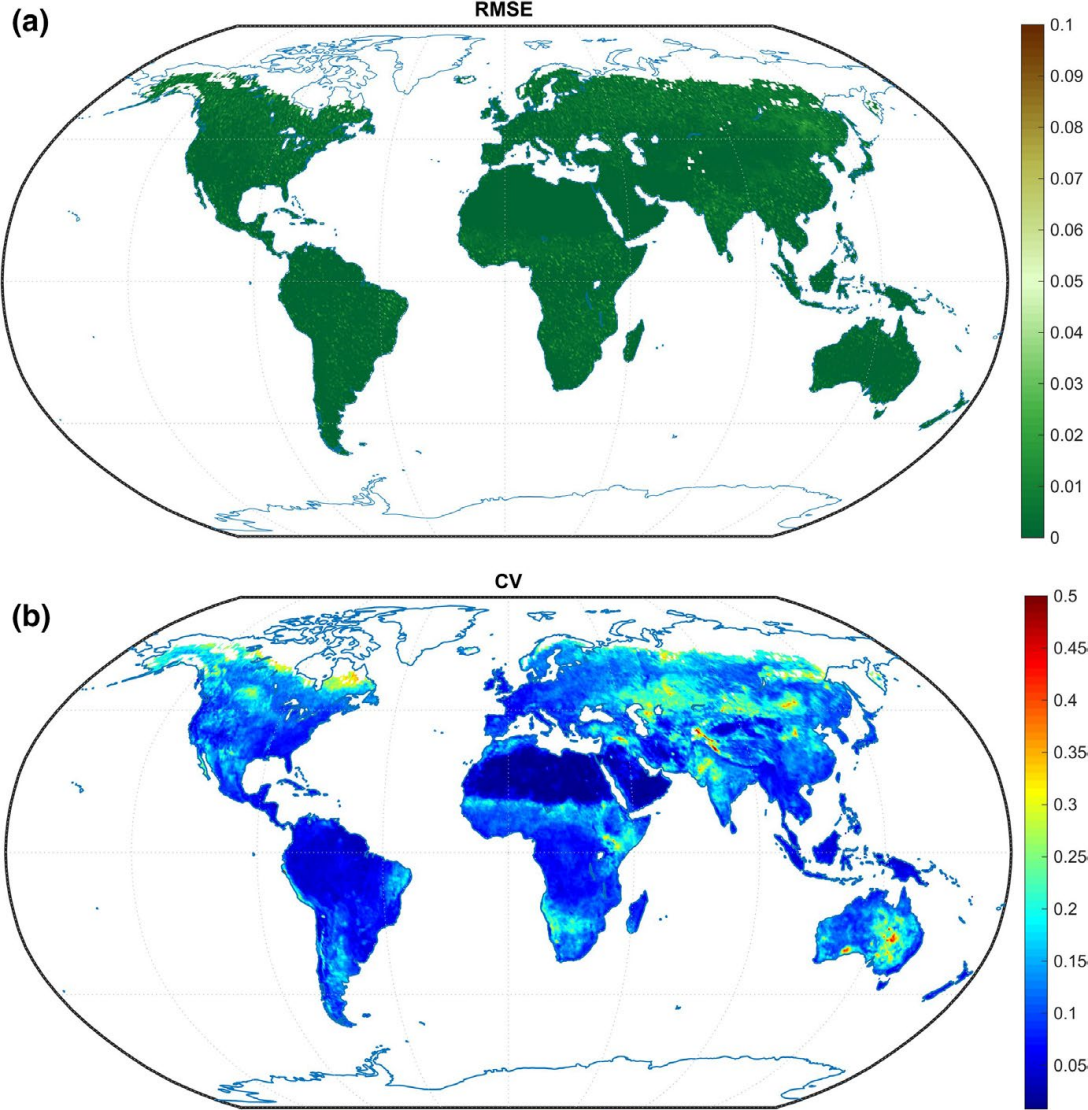
Statistics of model training and validation results. (a) represents the root mean square error (RMSE) to reveal the global model fitting accuracy on the training sets. (b) represents coefficient of variation (CV) to validate the models on the validating sets

Our results suggest that pixel‐level deep‐learning driven models are effective in simulating the dynamics of vegetation with enhanced performance (Figure 1). For model training, approximately 92% of the global areas have RMSE < 0.01 (Figure 2a). Due to the nonlinear modeling and multilevel expression of deep learning, the fitting precision of our models is very high. Results show that for 75% of the global gridded models, CV that measures the deviation of measured data from predicted data is <15% (Figure 2b).

Spatial differences exist in the predictive skill of the models. In general, the performance is better in the Southern Hemisphere than in the Northern Hemisphere. The regions with the best model performance (CV < 10%) are mainly located near the equator (in South America and Africa), while the regions with relatively poor model performance (CV > 20%) are mainly distributed in the boreal region of the Northern Hemisphere (including the Tibetan Plateau) (Figure 2b). Overall, most of the models established in our study are good at simulating and projecting vegetation greenness, which ensures our ability to predict future vegetation dynamics.

### 3.2 Sensitivity analysis

**FIGURE 3:**
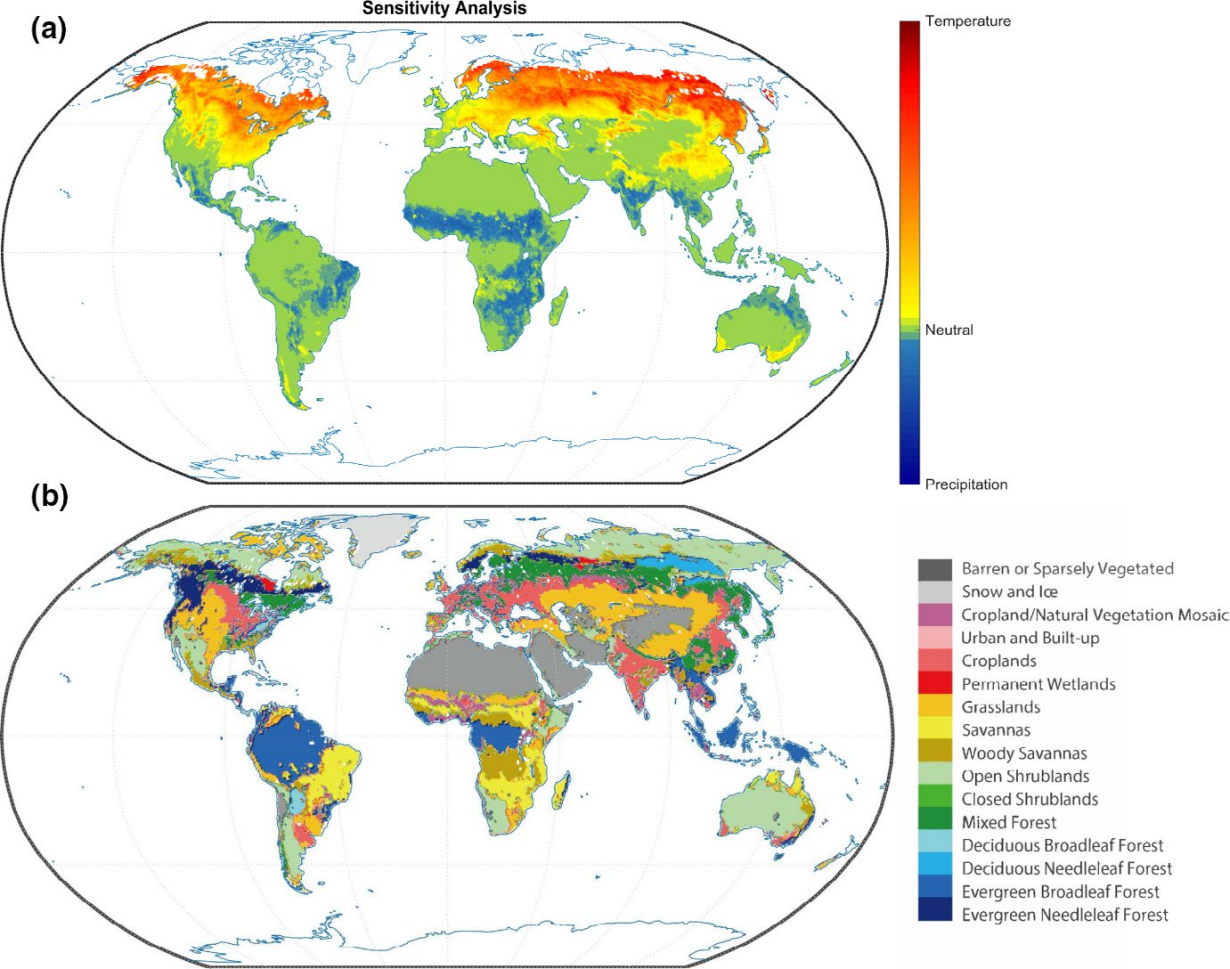
Sensitivity analysis of the models. (a) represents the global vegetation sensitivity to climate. (b) shows the spatial distribution of global land cover types based on the International Geosphere‐Biosphere Program (IGBP) scheme and the moderate resolution imaging spectroradiometer land cover product MOD12C1

Sensitivity analysis of models showed a spatial pattern of global vegetation to climate factors (Figure 3). The results show that 47.96% of the terrestrial ecosystem is more sensitive to temperature than it is to precipitation, especially at high latitudes in the Northern Hemisphere; this trend is also extended to Northeast China, central North America, and Southeast Australia (PID > 0.01). Approximately 18.48% of the globe is more sensitive to precipitation than to temperature (PID < −0.01) (Figure 3). These areas are mainly distributed in the tropical regions with savannas and grasslands. Our models provide a new method to analyze the sensitivity of global vegetation to climate based on data processing. Notably, the model performance in the areas equally sensitive to temperature and precipitation is better than that in the areas more sensitive to temperature or precipitation alone, which reminds us that vegetation sensitivity to temperature and precipitation can affect model accuracy. This suggests that the impacts of climate sensitivity on vegetation should be considered in the modeling of future vegetation dynamics.

At the biome level, the deciduous needleleaf forest (PIT = 0.134) and permanent wetlands (PIT = 0.088) are the most sensitive land cover types to temperature. And savannas (PTD = 0.011) is the most sensitive types to precipitation. We also found that deciduous needleleaf forest and permanent wetland mainly distributed in Northeast Asia had the greatest model errors, as well as vegetation interannual variation, with the indication that these vegetation respond to climate very complicatedly and vegetation sensitivity of climate may influence the vegetation response to climate.

### 3.3 Model error analysis

**FIGURE 4:**
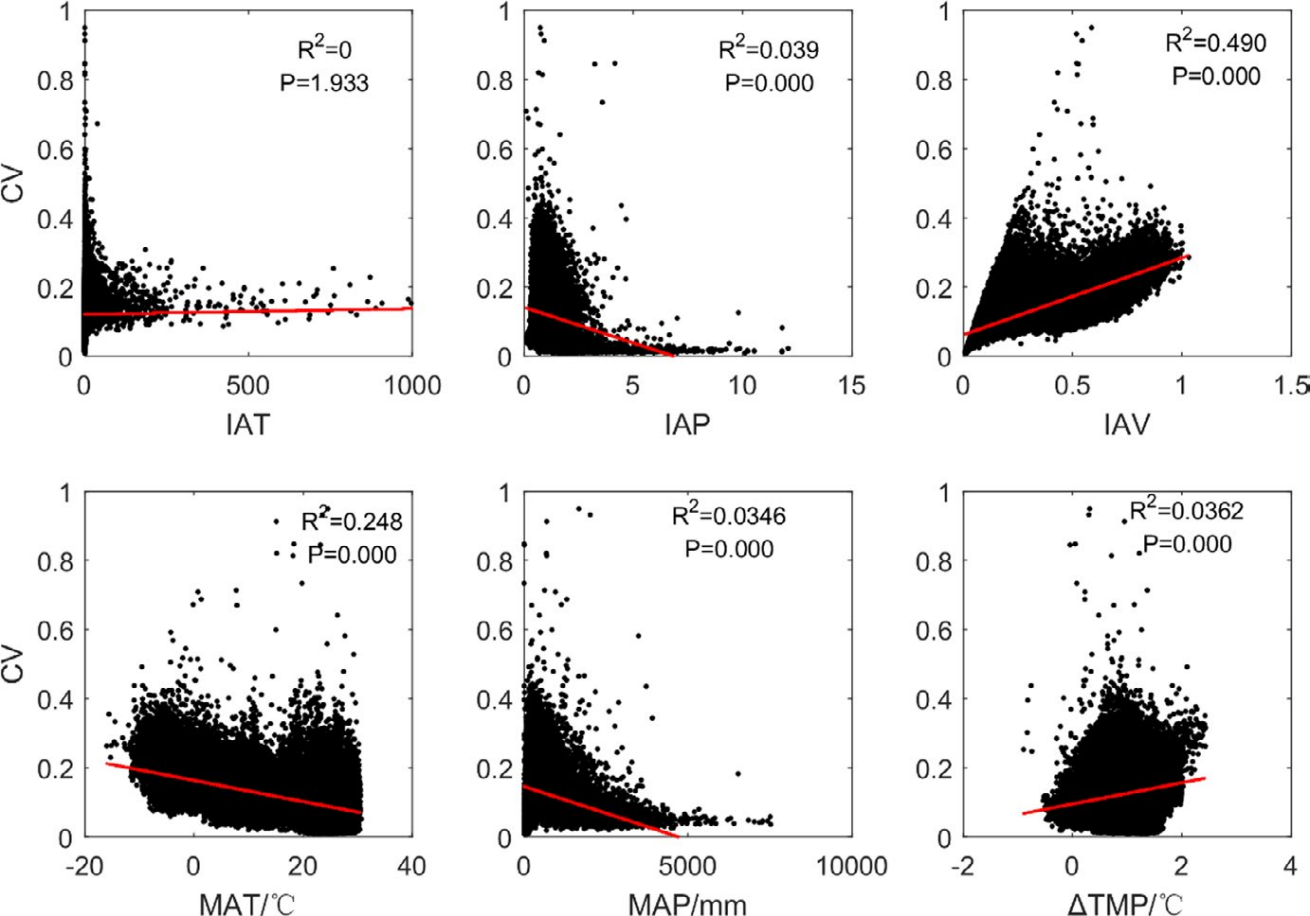
Correlations between the model CV and the related variables: (a) Interannual variations in temperature(IAT); (b) Interannual variations in precipitation(IAP); (c) Interannual variations in vegetation (IAV); (d) Mean annual temperature (MAT); (e) Mean annual precipitation (MAP); and (f) Increasing temperature (ΔTMP). All the values were calculated based on remotely sensed data from 1982 to 2015

**FIGURE 5:**
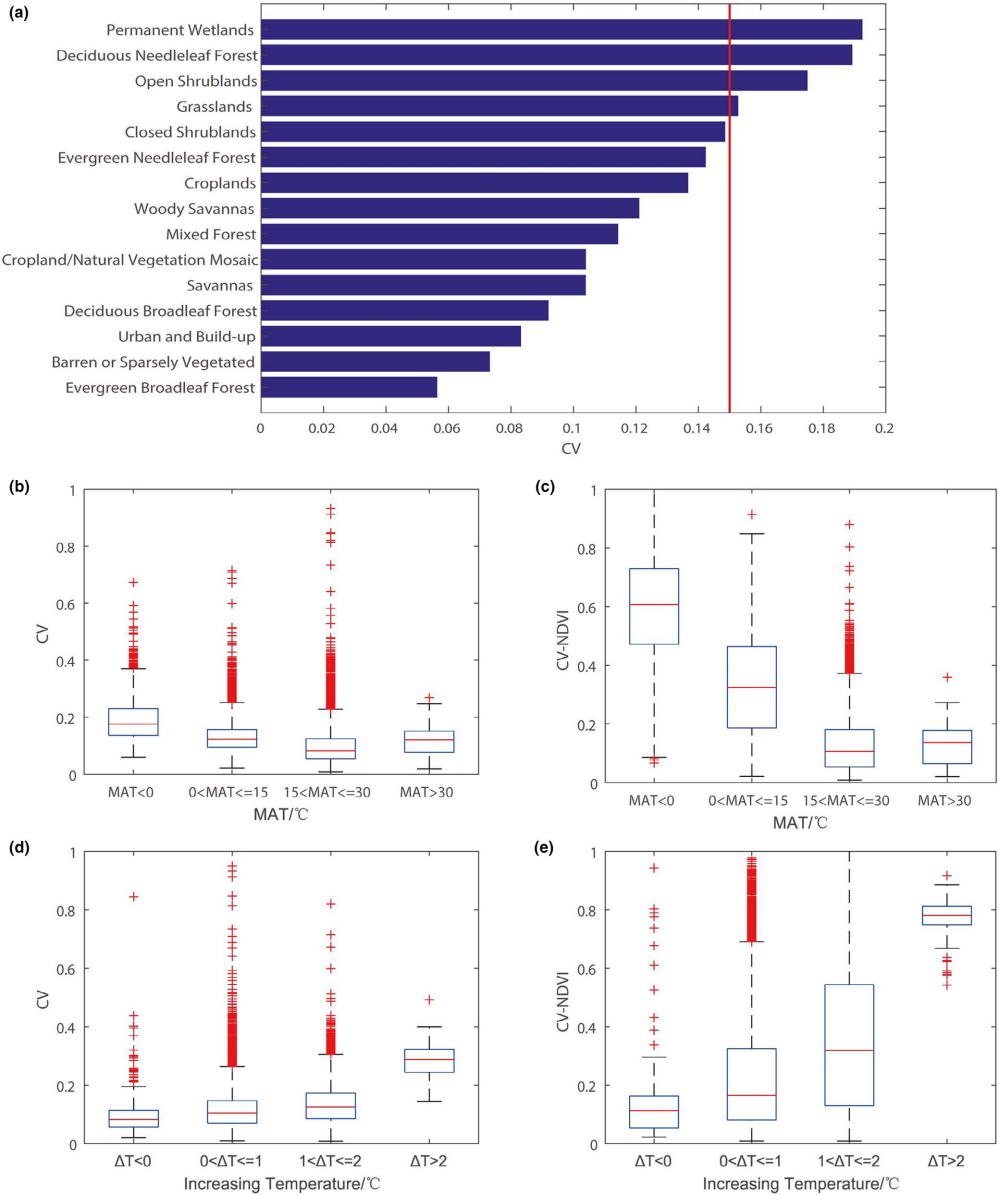
Model performance in different land cover types and temperature scenarios. (a) The model performances (CV) vary with land cover types (defined by the MODIS‐based IGBP land cover classification types for 2012). (a) CV > 15% means that the modeling accuracy is relatively low, and CV < 15% means a high modeling accuracy. The data used here are output by the model validation datasets. (b, c) The relationship between mean annual temperature (MAT) and model CV and the vegetation variation (IAV), calculated by the NDVI coefficient of variation from 1982 to 2015. (d, e) The relationship between temperature change (from 1982 to 2015) and model CV and the vegetation variation (IAV)

To understand the causes of model errors, we further analyzed the correlations between the model error CV and the related variables (Figure 4). Ultimately, we associated the model error with the interannual variation in vegetation (*R* ^2^ = 0.49, *p* = 0.00) (Figure 4). The model error is highly consistent with the vegetation interannual variation. For vegetation with smaller interannual variations, such as evergreen broadleaf forest and deciduous broadleaf forest, the performance of the models is very good (CV = 5%–9%) (Figure 5a). They are mainly distributed in areas where MAT > 15°C (Figure 5bc), temperature increase <0°C (Figure 5de), and sensitive to both temperature and precipitation (Figure 3). For vegetation types with larger interannual variations, such as permanent wetlands and deciduous needleleaf forest, our model performance is slightly worsened (CV = 17%–19%) (Figure 5a). They are mainly distributed in areas where MAT < 0°C (Figure 5bc), temperature increase >2°C (Figure 5de), and with significant sensitivity to temperature (Figure 3).

This indicates that in temperature‐restricted areas, the rising temperature in recent years may lead to the increase in vegetation volatility. The variability of vegetation is greatest in the region with warming of more than 2℃ and is much greater than in other regions, which reminds us that the increase of temperature in excess of 2°C may seriously affect vegetation growth. Moreover, there is a certain denotation in the model performance, that is, regions with poor model performance are likely to be regions with large interannual variations in vegetation, and these regions (especially the boreal permafrost regions and the highland vegetation in the Tibetan Plateau) may be more vulnerable to climate warming. Therefore, there should be more focus on and conservation action in these areas.

## 4 DISCUSSION

Climate factors are the most important variables affecting vegetation greenness, and the study of vegetation–climate relationship is a basis for simulating and predicting vegetation dynamics. Based on the vegetation–climate relationship, previous works have built many vegetation models at global scale, which are mostly based on the simple statistical relationships (Stephan et al., 2008; Hewson et al., 2019; Jorgenson et al., 2010; Lieth, 1975; Schuur, 2003; Zhu et al., 2016). The complex relationship of variables and dynamic processes and their interactions cannot be accurately simulated. Especially for vegetation response to the climate variation and adaptability, most of the vegetation dynamic models are based on the hypothesis that vegetation has a fixed pattern of response to climate change.

Deep‐learning techniques can capture small changes of vegetation response to climate patterns in the long‐time series, thus enhance the advantage and the accuracy of the vegetation model. What's more, deep learning has been proved to be an effective method to solve the complex relationship between variables, and LSTM models can efficiently predict the vegetation dynamic time series (Reddy & Prasad, 2018). In this research, we combine the big data platform and deep‐learning technology to achieve global pixel modeling, simulate vegetation over a long‐time scale. Sensitivity analyses of machine learning‐based models can help us thoroughly understand the relationship between different variables. Though our deep‐learning model is data‐driven and it was used to be seen as a black box with an insufficient ability to interpret mechanisms from the models, which now has been proved transparent and interpretable by various methods to understand the results (Lucas, 2020), it is very valuable and potential in helping us understand and project the nature.

Notably, though our models developed with LSTM are powerful with monitoring the vegetation dynamics, the performance of our model is slightly worsened in boreal regions and cannot describe thoroughly the relationship between the climate and vegetation especially for deciduous needleleaf forest and permanent wetland, which are the most sensitive biomes to temperature and with the highest interannual variability. It reminds us that these types of vegetation respond to climate very complicatedly and they may be more sensitive to future climate warming. It is also a common problem of current process‐based ecosystem models conducted in this region (Keenan & Riley, 2018; Pearson et al., 2013). Previous studies showed that vegetation growth has been found increasing rapidly during the past few decades in boreal regions (Mahowald et al., 2016; Myneni et al., 1997; Pearson et al., 2013). This is consistent with recent warming and resulted in the greening trend of high latitudes (Keenan & Riley, 2018; Zhu et al., 2016) along with the quick vegetation type shifts and uncertain climate feedbacks (Pearson et al., 2013). High latitude vegetation models are known to perform more poorly and tend to overestimate the vegetation growth extent and trend due to the elusive vegetation functional types and phenology (Anav et al., 2013; Mahowald et al., 2016; Murray‐Tortarolo et al., 2013). Thus, it still remains a challenge to better reveal and predict the northern vegetation growth patterns.

The significant temperature rising in recent years may partly explain the interannual variations in vegetation growth in boreal regions (Keenan & Riley, 2018; Nolan et al., 2018; Pearson et al., 2013), which is likely related to permafrost activities coupled with vegetation dynamics due to climate change (Jorgenson et al., 2010). Early research revealed that boreal region dynamics is dominated by mean annual air temperature, and rising temperature will lead to the melting of permafrost and the dissolution of underground ice (Vandenberghe et al., 2014). In recent years, the soil hydrothermal conditions in the northern permafrost region have undergone drastic changes with climate warming (Cazenave et al., 2009). Permafrost degradation also greatly affects surface water circulation and thus further influence the vegetation growth (Hinzman et al., 2005). The interannual fluctuations of vegetation growth increase, and the hydrothermal regulation of vegetation growth is unbalanced, which further leads to the continuous increase of interannual fluctuations of vegetation growth, increasing the instability of the regional ecosystem.

Besides the climate factors, elevated atmospheric CO_2_ concentration, varying rates of nitrogen deposition, land use, and other anthropic factors could also influence the vegetation greenness, which may bring a greater vegetation change potential due to the more complex factors interacted together (Zhu et al., 2016). Further, we can explore the more complex social–ecological systems by inputting more natural and anthropogenic variables and coupling with certain physical process based on deep learning to better understand the complex relationship of vegetation and the environment. And sensitivity analysis of machine learning can also help us investigate the sensitivity of vegetation to different variables through data mining, thus further consider the vegetation sensitivity and adoption to the environment into the modern vegetation dynamic models.

## 5 CONCLUSIONS

In summary, we apply state‐of‐the‐art technology (i.e., deep learning) to build global gridded vegetation–climate models based on dynamic time series modeling. We conclude that deep learning is an effective way to simulate the long‐term vegetation greenness dynamics and investigate the climate sensitivity of vegetation. Our methods show that deep learning has a great potential in modeling long‐term vegetation dynamics. We achieved global gridded long‐time series modeling and effective sensitivity analysis to reveal vegetation response to climate change, which is a totally new attempt to integrate application of deep learning with big data to our ecological modeling studies, and it proves to be possible and necessary in the future under the context of big data and automatic monitoring. Further integrating more natural and anthropogenic factors in vegetation dynamics coupling with other physical models may yield a more reliable modeling result. More interpretable methods can also be used to improve the deep‐learning applications in ecology widely.

## CONFLICT OF INTEREST

None declared.

## AUTHOR CONTRIBUTIONS

**Zhiting Chen:** Data curation (equal); Formal analysis (equal); Investigation (equal); Writing‐original draft (equal). **Hongyan Liu:** Funding acquisition (equal); Methodology (equal); Project administration (equal); Supervision (equal); Writing‐review & editing (equal). **Chongyang Xu:** Investigation (equal); Writing‐review & editing (equal). **Xiuchen Wu:** Investigation (equal); Writing‐review & editing (equal). **Boyi Liang:** Investigation (equal); Writing‐review & editing (equal). **Jing Cao:** Investigation (equal); Writing‐review & editing (equal). **Deliang Chen:** Writing‐review & editing (equal).

## Data Availability

All the data used in this paper are available online. Gimms NDVI 3g data: https://climatedataguide.ucar.edu/climate‐data/ndvi‐normalized‐difference‐vegetation‐index‐3rd‐generation‐nasagfsc‐gimms. Climate data: http://badc.nerc.ac.uk/browse/badc/cru. Land cover product MOD12C1: http://glcf.umd.edu/data/lc/. Permutation importance was implemented in Python through the eli5 package: https://pypi.org/project/eli5/.
